# PRE-REGISTERING QUALITATIVE RESEARCH: BENEFITS, UNINTENDED CONSEQUENCES, AND UNANSWERED QUESTIONS

**DOI:** 10.1093/geroni/igz038.1483

**Published:** 2019-11-08

**Authors:** Barbara Bowers

**Affiliations:** University of Wisconsin-Madison, School of Nursing, Madison, Wisconsin, United States

## Abstract

While preregistration has gained increasing acceptance for quantitative, particularly experimental, studies, its relevance and implementation for qualitative research has only recently been proposed. This presentation provides an overview of the very recent and ongoing debate on the potential benefits and costs of implementing preregistration for qualitative research. The presentation summarizes the debates about whether and how preregistration will lead to greater transparency in qualitative research, explores the implications of preregistration for qualitative research, identifies some of the costs incurred in preregistering qualitative studies, describes the inaccurate assumptions about qualitative research that are repeatedly embedded in calls for preregistration, and identifies some likely, unintended consequences of adopting the same approaches employed or proposed for quantitative studies. Acknowledging the importance of greater transparency and reduced publication bias for all research, including qualitative studies, questions about transparency that qualitative researchers must urgently address are also suggested.

